# A Chinese Herbal Formulation, Xiao-Er-An-Shen Decoction, Attenuates Tourette Syndrome, Possibly by Reversing Abnormal Changes in Neurotransmitter Levels and Enhancing Antioxidant Status in Mouse Brain

**DOI:** 10.3389/fphar.2019.00812

**Published:** 2019-07-24

**Authors:** Jihang Chen, Pou Kuan Leong, Hoi Yan Leung, Wing Man Chan, Zhonggui Li, Jingyu Qiu, Kam Ming Ko, Jianping Chen

**Affiliations:** ^1^School of Life and Health Science, The Chinese University of Hong Kong, Shenzhen, China; ^2^Division of Life Science, Hong Kong University of Science & Technology, Hong Kong, China; ^3^Shenzhen Key Laboratory of Hospital Chinese Medicine Preparation, Shenzhen Traditional Chinese Medicine Hospital, Guangzhou University of Chinese Medicine, Shenzhen, China

**Keywords:** Tourette sydrome, antioxidant status, Xiao-Er-An-Shen Decoction, neurotrasmitters, brain

## Abstract

Xiao-Er-An-Shen Decoction (XEASD) has been used clinically for the treatment of Tourette syndrome (TS) in children for more than 20 years in mainland China. The biochemical mechanism underlying the therapeutic action produced by XEASD treatment against TS remains unknown. However, a previous study has shown that pre-incubation of PC12 neuronal cells with XEASD can induce neurite outgrowth and protect against oxidative stress. In the present study, using a mouse model of TS induced by 3,3’-iminodipropionitrile (IDPN), stereotypy scoring, and locomotor activity were assessed. Levels of neurotransmitters including glutamate, aspartate, and gamma-aminobutyric acid (GABA) in brain tissue as well as plasma cyclic adenosine monophosphate (cAMP) were measured using assay kits. The ratio of reduced glutathione (GSH)/oxidized glutathione (GSSG) and Mn-superoxide dismutase (MnSOD) activity in brain mitochondrial fractions as well as mitochondrial glutathione reductase and cytosolic γ-glutamylcysteine activities were also examined. The phosphorylation of cAMP-responsive element binding protein (CREB) in brain tissue was measured by Western blot analysis. XEASD treatment was found to significantly ameliorate the severity of behavioral symptoms in affected mice, as evidenced by decreases in the stereotypy score and locomotor activity. The beneficial effect of XEASD was accompanied by the reversal of abnormal levels of GABA, glutamate, and aspartate, in brain tissue of IDPN-challenged mice. In addition, XEASD treatment increased plasma cyclic adenosine monophosphate (cAMP) levels and activated the phosphorylation of CREB in brain tissue of TS mice. Furthermore, XEASD treatment was found to enhance the antioxidant status of brain tissue in affected mice, as evidenced by increases in the GSH/GSSG ratio and the activity of MnSOD in brain mitochondrial fractions. Taken together, these experimental results will hopefully provide insight into the pharmacological basis for the beneficial effects of XEASD in children suffering from TS.

## Introduction

Tourette syndrome (TS) is a neuropsychiatric disorder characterized by involuntary and repetitive, habitual, non-rhythmic, and burst-like vocal/phonic and motor tics (Ji et al., 2016). It is a childhood-onset condition affecting approximately 1% of school-age children, boys being about three-to-four times more likely than girls to develop the condition. TS patients often show unpleasant premonitory urge sensations preceding tics that are relieved by their execution. Most TS patients also present with other behavioral and emotional conditions, such as symptoms of attention-deficit hyperactivity disorder, obsessive-compulsive disorder, depression, and anxiety.

Currently, it is widely accepted that the development of TS is causally related to an interaction among genetic, biochemical, immunological, and environmental factors (Martino et al., 2009). In terms of its pathogenesis, TS is thought to arise as the result of fundamental alterations in functional dynamics of cortico-striato-thalamo-cortical (CSTS) pathways (Nordstrom et al., 2015), which are also relevant to other hyper- and hypo-kinetic movement disorders, such as Parkinson’s disease and Huntington’s disease (Honey et al., 2003). Malfunctioning of these circuits may underlie the occurrence of tics. TS has been found to be closely associated with abnormalities in the neurotransmission of γ-aminobutyric acid (GABA) and glutamate, two major neurotransmitters within these neural circuits (Mahone et al., 2018). GABA regulates brain excitability *via* its GABA-A receptors. The inhibition of GABA signaling in striatal medium-sized spiny neurons and interneurons found within the striatum is thought to play a key role in the pathogenesis of TS. *Postmortem* examination of brains of individuals with TS has demonstrated the involvement of striatal GABAergic system, which includes substantial decreases of parvalbumin-containing interneurons and altered GABAA receptor binding within the striatum, the pathogenesis (Kataoka et al., 2010). Glutamate, which is the primary excitatory neurotransmitter of the central nervous system, is an essential component of neuronal pathways implicated in TS, and it is an extensive modulator of dopamine, the major neurotransmitter associated with tics. The involvement of glutamatergic system dysfunction within CSTS pathways was evidenced by reduced glutamate levels in *postmortem* brains of TS patients, especially in the globus pallidus interna, externa, and substantia nigra pars reticulate (Anderson et al., 1992). In addition, familial genetic studies showed that a missense mutation in the glial glutamate transporter gene is present in TS patients (Paschou et al., 2013).

In addition to the developmental disorder of synaptic transmission in the CSTS circuit, an increase in oxidative stress has been suggested to be involved in the pathogenesis of TS and other movement disorders. Abnormally high levels of biochemical indices of oxidative stress in blood samples were observed in children with TS (Landau et al., 2012). It is well known that mitochondria are both the primary sources of reactive oxygen species (ROS) and a major target of ROS-induced damage. In view of the finding that oral administration of a synthetic free radical scavenger, MCI-186, can alleviate some symptoms of TS in a mammalian model of the disease, reduction of oxidative stress might prove to be effective in the treatment of TS (Watanabe et al., 2008). In this regard, the glutathione antioxidant response, which is the pivotal mechanism of antioxidant defense against ROS and electrophiles, may also play an important role in mitigating the development of TS.

Within the framework of TCM theory, TS is considered to be closely associated with a syndrome arising from the propulsion of *Liver Wind* and an increase in phlegm formation, which are caused by excessive *Liver Yang* and *Spleen deficiency*, respectively (Lee, 2017). The agitated *Liver Wind* and increased phlegm formation by the *Spleen* act together to obstruct the transportation of *Qi* and thus harass *Shen* (which is closely related to brain function), resulting in the manifestation of symptoms of head jerking, shoulder shrugging, blinking eyes, winking eyebrows, pointing lips, yelling, kicking legs, irritability, and so on. Xiao-Er-An-Shen Decoction (XEASD), a Chinese herbal formulation consisting of nine individual herbs—namely, *Polygalae* Radix (the root of *Polygala tenuifolia*), *Astragali* Radix (the root of *Astragalus membranaceus*), *Acori* *tatarinowii* Rhizoma (rhizome of *Acorus tatarinowii* Schott), *Citri reticulatae pericarpium* (*Citrus reticulata* Blanco), *Alpiniae oxyphyllae* Fructus (fruits of *A. oxyphyllae*), *Aurantii* Fructus (fruits of *Citrus aurantium* L)., *Pinelliae* Rhizoma (rhizome of *Pinellia ternata* (Thunb)., *Notopterygii* Rhizoma *et* Radix (radix of *Notopterygium incisum* Ting ex H. T. Chang), and *Glycyrrhizae* Radix *et* Rhizoma (radix and rhizome of *Glycyrrhiza uralensis* Fisch) (Li et al., 2018)—has been clinically prescribed for the treatment of TS and other excitatory movement disorders in children for more than 20 years in mainland China. It is effectively used to provide nutrients to the brain and tranquillize and dispel *phlegm* to reduce fright in the practice of traditional Chinese medicine (TCM). The formulation of XEASD is based on the compatibility theory of herbal formulation in TCM wherein the “Chief” herb, *Acori tatarinowii* Rhizoma, acts primarily by eliminating *phlegm* and inducing a smooth flow of *Qi*. *Polygalae* Radix, *Astragali* Radix, *A. oxyphyllae* Fructus, *Aurantii* Fructus, and *Pinelliae* Rhizoma serve as “adjuvant” herbs, which can invigorate *Spleen Qi*, eliminate *phlegm*, and suppress *Liver Wind*. The nine herbs of XEASD, when acting together, can resolve the *phlegm*, settle *Liver Wind*, and nourish *Shen*, thereby effectively alleviating symptoms of TS in clinical conditions. Our previous study in humans has demonstrated that children with TS showed a decreased frequency of involuntary movements and vocalizations after XEASD treatment, when compared with those receiving haloperidol treatment (Qiu Jingyu et al., 2010). However, the biochemical mechanism(s) underlying the beneficial effects of XEASD in TS remained largely unknown. In the present study, we investigated the effects of XEASD in a mouse model of TS induced by 3,3’-iminodipropionitrile (IDPN) by assessing the grades of stereotypy and locomotor activities. The levels of several neurotransmitters as well as antioxidant status in brain tissues were also measured to explore the possible biochemical mechanism(s) underlying the beneficial effect of XEASD in TS.

## Materials and Methods

### Materials

XEASD was provided by Pharmaceutical Department of Shenzhen Traditional Chinese Medicine Hospital. Caffeic acid (1), morroniside (2), loganin (3), liquiritin (4), tenuifolin (5), calycosin-7-O-b-glucoside (6), ammonium glycyrrhizate (7), naringin (8), 3,6’-disinapoyl sucrose (9), hesperidin (10), neohesperidin (11), astragaloside IV (12), and isoimperatorin (13) were purchased from National Institutes for Food and Drug Control (Beijing, China). The purities of all these chemicals were over 98%. HPLC grade acetonitrile was purchased from Merck (Darmstadt, Germany), and ultrapure water was obtained from a Milli-Q purification system (Molsheim, France).

### Chromatographic Conditions and Instrumentation

A validation HPLC method was applied onto a Shimadzu (Kyoto, Japan) LC-20AT system. The herbal extract was separated on Agilent ZORBAX SB-C18 (250 mm × 4.6 mm, 5 μm) column. The mobile phase was composed of 10 mmol/ml ammonium acetate solution (A) and acetonitrile (B) using the following gradient program: 0.01–22 min, 15–35%B; 22.1–35min, 90%B; the flow rate was 0.8 ml/min; the injection volume was 5 μl. A Shimadzu mass spectrum (LC-2020) equipped with an ESI ion source was performed in both positive and negative modes, and the selected ion monitoring (SIM) was employed. Optimized mass spectra were acquired with an interface voltage of 4.5 kV. Nitrogen was used as nebulizer gas at a flow rate of 1.5 L/min and dry gas flow of 15 L/min. Shimadzu Mass Workstation Software was used for data acquisition and processing.

### Animal Treatment

Male ICR mice (4–7 weeks, 18–20 g) were randomly assigned to six groups, with 5–10 animals in each. Mice in the control group received an intraperitoneal injection of normal saline (NS) (0.9%) at a dose of 0.3 ml/day. In the IDPN-challenged groups, mice were injected with IDPN (450 mg/kg/day) intraperitoneally for 4 days; 24 h after administering the last dose of IDPN, mice in the challenged groups were administered NS intragastrically at 10 ml/kg/day (i.e., control group), or tiapride hydrochloride (Tia) at 100 mg/kg/day (positive control group) for 4 days followed by 10 days with NS, or XEASD at increasing doses (2, 4, and 8 g/kg; XEASD groups) for 14 days by gavage. All experimental protocols were reviewed and approved by the University Ethics Committee on Animal Research Practice at the Hong Kong University of Science and Technology.

### Behavioral Assessments

Grades of stereotypy were evaluated by observing the behavior of IDPN-challenged mice, with or without treatment, as described by Wang et al. (2013)(**Table 1**).

**Table 1:** Behavior assessment with reference to grades of stereotypy.

Score	Stereotypy
0	No stereotypy or normal activity
1	Discontinuous circling behavior (clockwise/counterclockwise circling)
2	Occasional head twitching Occasionally vertical dyskinetic head and neck movements
3	Occasional sniffing, licking, and biting Continuous circling behavior, increased body raising
4	Increased sniffing, repetitive grooming (such as paw-to-mouth movements) Increased lateral and vertical dyskinetic head and neck movements

### Locomotor Activity Test

Locomotor activity was measured in a fully computerized multi-box infrared-sensitive motion-detection system, as described in Wei et al. (2005). The apparatus consisted of four cylindrical compartments (diameter: 25 cm; height: 13 cm) made of transparent Lucite. The four compartments, visually blocked from each other, were put inside a large black opaque plastic box. For each compartment, three pairs of sending–receiving photoelectric cells were placed around the side, 1 cm above the bottom of the box, forming three light beams traveling through the box at an angle of 60° between every two light beams. Horizontal locomotor activity was measured as mice moved, interrupting the light beams, thereby generating signals that were sent to the computer for quantifying the activity. One mouse was placed in each box, and four mice were tested simultaneously. The total number of signals recorded within 5 min was used as an index of locomotor activity. All mice were subjected to the locomotor activity test (LAT) for 2 days, once per day.

### Measurement of Neurotransmitter Levels in Brain Tissue

Levels of neurotransmitters including glutamate, aspartate, and GABA in brain tissue were measured.

For the measurement of glutamate levels in brain tissue, brain homogenate samples were incubated with mammalian cell lysis buffer (PerkinElmer) at a 1:1, v/v ratio, and further lysed by shearing mechanical force achieved by passing the mixture through an 18- or 21-gauge needle. The lysates were centrifuged at 2,150 ×*g* at 4°C for 10 min, and supernatants were analyzed using a Glutamate Assay Kit (Fluorometric) (Abcam).

For the measurement of aspartate levels in brain tissue, brain homogenate samples were combined with 0.1 M HCl at a 1:1 v/v ratio. The mixtures were centrifuged at 2,150 ×*g* at 4°C for 10 min, and supernatants were analyzed using an aspartate Colorimetric/Fluorometric Assay Kit (BioVision).

For the measurement of GABA levels in the striatum of the brain, striatal tissue was isolated from the whole brain and subjected to homogenization in 0.7 ml of mammalian cell lysis buffer. The samples were further lysed by shearing mechanical force achieved by passage through an 18- or 21-gauge needle. The lysates were centrifuged at 20,000 ×*g* at room temperature for 5 min, and supernatants were used for the measurement using Mouse GABA ELISA Kit (MyBioSource).

### Measurement of Cyclic Adenosine Monophosphate in Plasma

Plasma samples were prepared from whole blood samples treated with an anticoagulant, heparin, and used to measure cyclic adenosine monophosphate (cAMP) using a direct immunoassay kit (colorimetric) according to the manufacturer’s instruction of the assay kit (BioVision).

### Measurement of cAMP Response Element (CREB) Levels in Brain Tissue

Western blot analysis was used to detect p-CREB and total CREB levels in brain tissues. Equal amounts of nucleus-free fractions of brain tissue were mixed with Laemmli’s loading buffer, and the mixture was boiled for 5 min and then separated using 10% SDS-PAGE at 120 V, followed by electroblotting to nitrocellulose membranes (Bio-Rad, CA, USA) for 2 h at 100 V. Membranes were blocked for 1 h with 5% nonfat milk in TBS-T (50 mM Tris, 150 mM NaCl, 0.05% Tween-20) at room temperature. The membranes were incubated at 4°C with antibodies for p-CREB (phospho S133) or CREB (1:500, Abcam) overnight. The membranes were rinsed with TBS-T and incubated with a horseradish-peroxidase-conjugated secondary antibody (1:4,000, Santa Cruz, CA). After the incubation, the membranes were rinsed with TBST, and the immuno-stained protein bands were quantitatively analyzed by densitometry using an ECL Western Blot System (Cell Signaling Technology, Beverly, MA) following the manufacturer’s instructions.

### Measurement of GSH/GSSG Ratio and the Activity of MnSOD in Brain Mitochondrial Fractions

The mitochondrial reduced glutathione (GSH)/oxidized glutathione (GSSG) ratio and the activity of manganese-superoxide dismutase (MnSOD) were measured by enzymatic and spectrophotometric methods, respectively, as previously described (Ko et al., 2010; Chen and Ko, 2013).

### Measurement of Mitochondrial GR and Cytosolic GCL Activity in Mouse Brain Tissues

Mitochondrial glutathione reductase (GR) and cytosolic γ-glutamylcysteine (GCL) activities were measured by monitoring the oxidation of NADPH spectrophotometrically as previously described (Lam and Ko, 2011).

### Statistical Analysis

All statistical analyses were performed using SPSS 16.0 (SPSS Inc., Chicago, IL, USA). Data shown in the figures and tables are mean ± SD or mean ± SEM, and they were analyzed by one-way analysis of variance (ANOVA). *Post hoc* multiple comparisons were performed using Tukey or Dunnett T3 tests, depending on the results of the homogeneity of variance test (Levene’s test). Differences were considered statistically significant for *P* < 0.05.

## Results

### LC-MS Analysis of XEASD Extract

**Figure 1:**
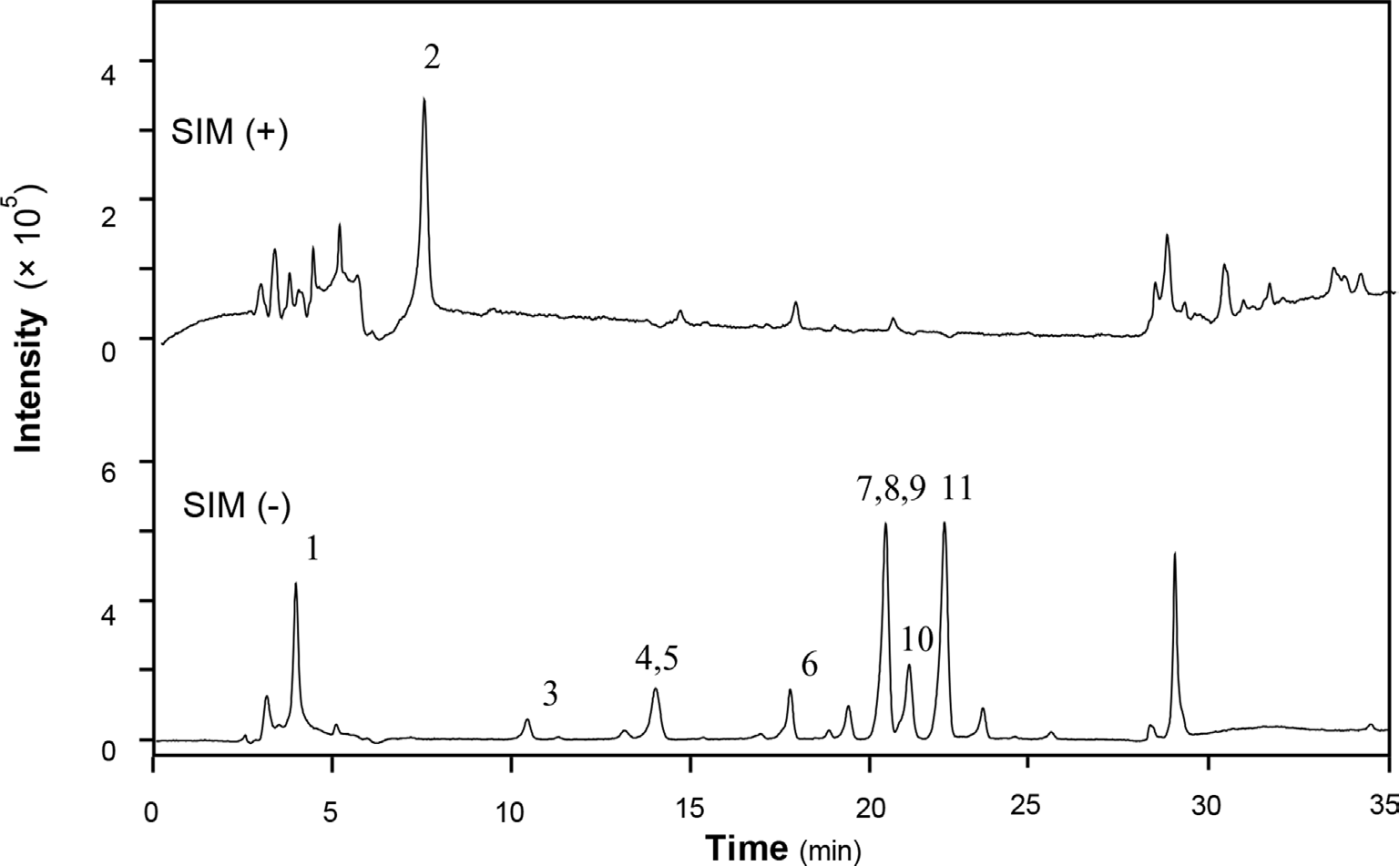
HPLC-MS chromatogram of XEASD extract. A representative LC-MS chromatogram from three batches of XEASD extract is shown. Eight chemical markers were identified in the XEASD extract. The denotation peaks 1–13 were caffeic acid (1), morroniside (2), loganin (3), liquiritin (4), tenuifolin (5), calycosin-7-O-β-glucoside (6), ammonium glycyrrhizate (7), naringin (8), 3,6’-disinapoyl sucrose (9), hesperidin (10), neohesperidin (11), astragaloside IV (12), and isoimperatorin (13).

XEASD was chemically analyzed for standardization. An high performance liquid chromatography–mass spectrometry (HPLC-MS) method was established to reveal the chemical profile of XEASD extract and quantify the main ingredients in the extract. Thirteen compounds were identified from XEASD extract (**Figure 1**), and the minimum amount in mg/g of dried extract was 0.9975 for caffeic acid, 0.5869 for morroniside, 0.4474 for loganin, 0.1881 for liquiritin, 0.2167 for tenuifolin, 0.3330 for calycosin-7-O-β-glucoside, 0.3121 for ammonium glycyrrhizate, 1.509 for naringin, 0.1722 for 3,6’-disinapoyl sucrose, 0.1810 for hesperidin, 0.6390 for neohesperidin, 0.0380 for astragaloside IV, and 0.6903 for isoimperatorin.

### Behavioral Assessment

#### Stereotypy Scoring

As shown in **Table 2**, IDPN challenge dramatically increased the stereotypy score in mice by 836%. Most of the IDPN challenged mice showed obvious continuous circling behavior and body raising. Using one-way ANOVA, we found that both Tia and XEASD treatments significantly reduced the scores (by 42%, *P* < 0.01 and 75–82%, *P* < 0.01, respectively), when compared with the untreated TS mice. Tia-treated mice mainly showed discontinuous circling behavior, while XWASD treatment at various doses showed no obvious stereotypy.

**Table 2:** Effects of Tia and XEASD treatments on stereotypy scoring in IDPN-induced TS mice.

Group	Stereotypy scoring
Control 1	0.023 ± 0.016
IDPN (450 mg/kg)	
Control 2	1.947 ± 0.099^*^
Tia (100 mg/kg)	1.135 ± 0.133^*#^
XEASD (2 g/kg)	0.343 ± 0.081^*#^
XEASD (4 g/kg)	0.497 ± 0.122^*#^
XEASD (8 g/kg)	0.394 ± 0.090^*#^

Grades of stereotypy were evaluated as described in Materials and Methods. Control 1 represents normal control mice; control 2 represents IDPN-challenged control mice. Data are shown as the mean ± SEM, with n ≥ 23.*P < 0.05, significantly different from the control 1; ^#^P < 0.05, significantly different from the IDPN control 2.

#### Locomotor Activity

**Figure 2:**
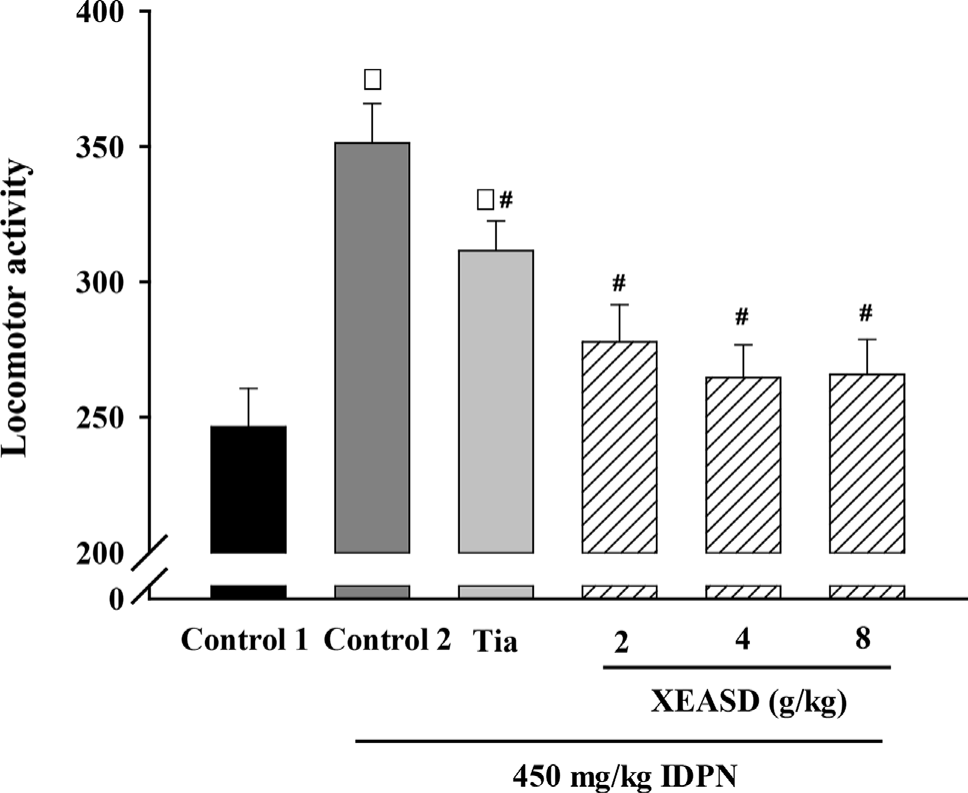
Effects of XEASD treatment on locomotor activity of IDPN-induced TS mice. Locomotor activity was measured as described in Materials and Methods. Control 1 represents normal control mice; control 2 represents IDPN-challenged control mice. Each bar represents mean ± SEM, with *n* ≥ 23. *P < 0.05, significantly different from the control 1; ^#^ *P* < 0.05, significantly different from IDPN control 2. Locomotor activity of control 1 (mean ± S.E.M). = 247 ± 14.1.

We examined whether the behavioral changes induced by Tia or XEASD were accompanied by variations in locomotor activity. **Figure 2** shows that IDPN-injected mice showed a 43% increase in locomotor activity, as assessed by measuring the number of interruption on light beams by the moving mouse. Treatment with Tia (100 mg/kg) or XEASD (2, 4, 8 g/kg) significantly decreased locomotor activity by 11% (*P* < 0.05) and 21% (P < 0.01) and 25% (*P* < 0.01) and 24% (*P* < 0.01), respectively, when compared with the untreated TS animals.

#### Effects of XEASD Treatment on Neurotransmitter Levels in Brain Tissue of TS Mice

**Figure 3:**
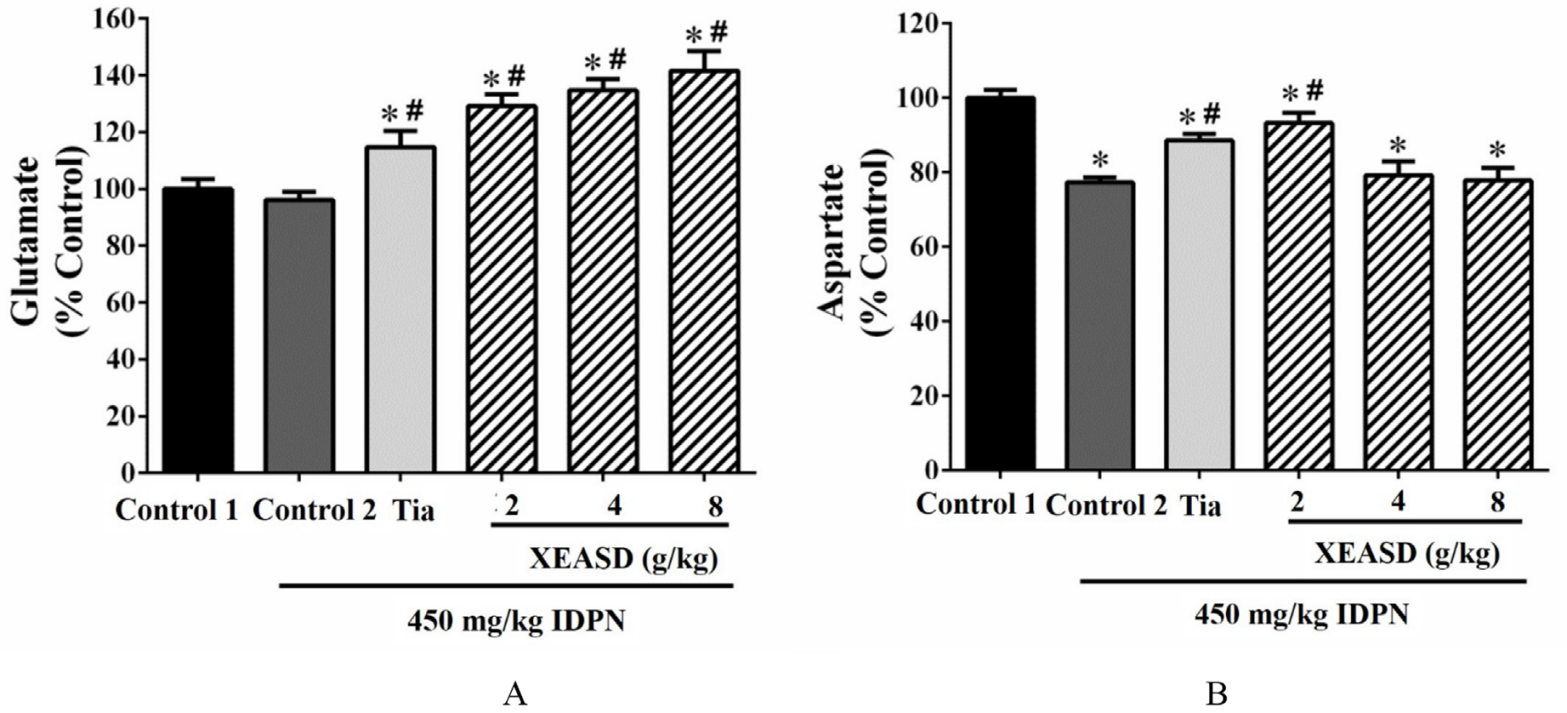
Effects of XEASD treatment on glutamate and aspartate levels in brain tissue of IDPN-induced TS mice. **(A)** Biochemical assay of glutamate levels was performed as described in Materials and Methods. **(B)** Levels of aspartate in brain tissues were measured as described in Materials and Methods. Control 1 represents normal control mice; control 2 represents IDPN-challenged control mice. Data are expressed as the percentage of non-challenged control 1 values. *P < 0.05, significantly different from the control 1; ^#^ *P* < 0.05, significantly different from the IDPN control 2. The value of the glutamate levels of control 1 (mean ± S.E.M.) and aspartate level of control 1 (mean ± S.E.M.) were 2953 ± 440 (nmol/mg protein) and 0.73 ± 0.06 (nmol/mg protein), respectively.

As depicted in **Figure 3A**, the ANOVA revealed that whereas IDPN challenge caused a small but insignificant decrease in brain glutamate levels, both Tia and XEASD treatments significantly increased glutamate levels (15−42%, *P* < 0.01), when compared with the untreated TS mice.

Brain aspartate levels were significantly decreased (by 23%, *P* < 0.01) in TS mice, when compared with the normal control (**Figure 3B**). Both Tia and XEASD (2 g/kg) significantly increased aspartate levels in TS mice (15%, *P* < 0.01 and 21%, *P* < 0.01, respectively), when compared with the untreated TS mice.

#### GABA Levels in the Striatum

**Figure 4:**
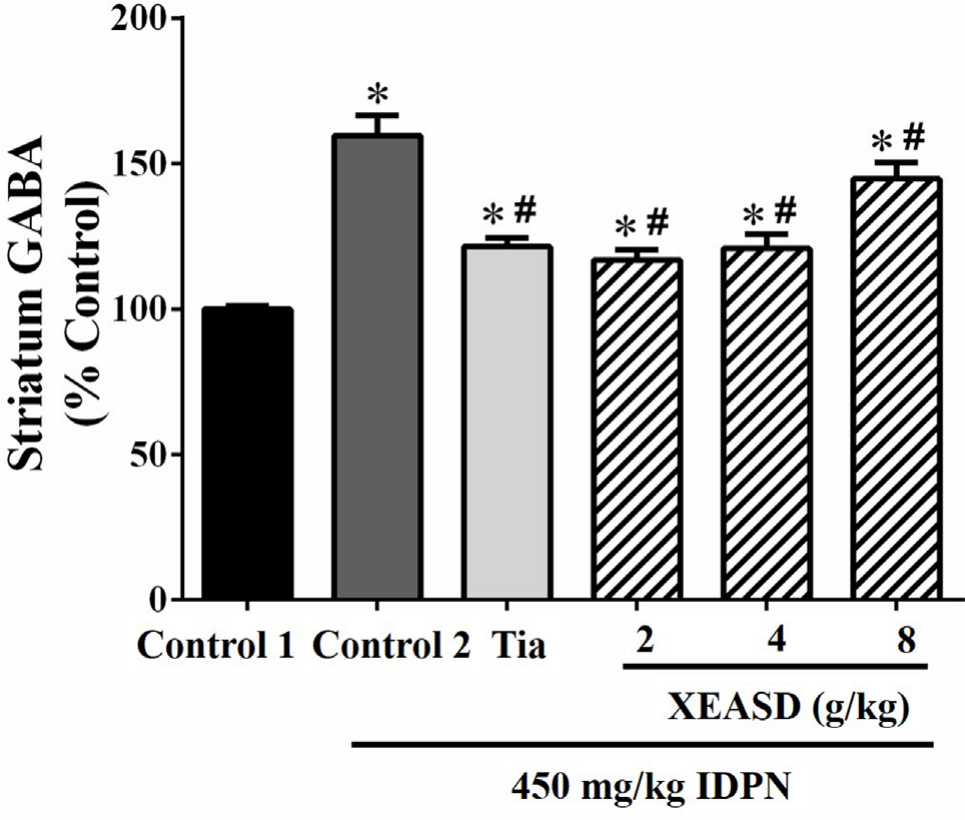
Effects of XEASD treatment on gamma-aminobutyric acid (GABA) levels in brain striatum of IDPN-induced TS mice. Biochemical assay of striatal GABA levels was performed as described in Materials and Methods. Control 1 represents normal control mice; control 2 represents IDPN-challenged control mice. Data are expressed as the percentage of non-challenged control 1 values. *P < 0.05, significantly different from the control 1; ^#^ *P* < 0.05, significantly different from the IDPN control 2. The value of GABA level of control 1 (mean ± S.E.M.) = 2.03 ± 0.15 (pg/mg protein).

IDPN challenge significantly increased GABA levels (60%, *P* < 0.01) in the striatum of mice in the model group, when compared with the normal animals (**Figure 4**). Both Tia and XEASD treatments (2 and 4 g/kg) significantly reduced GABA levels (by 24−27%, *P* < 0.01) in TS mice, when compared with the IDPN-challenged TS controls. XEASD treatment at the dose of 8 g/kg also significantly decreased GABA levels by 9% (*P* < 0.05).

#### Plasma cAMP Levels in TS Mice

**Figure 5:**
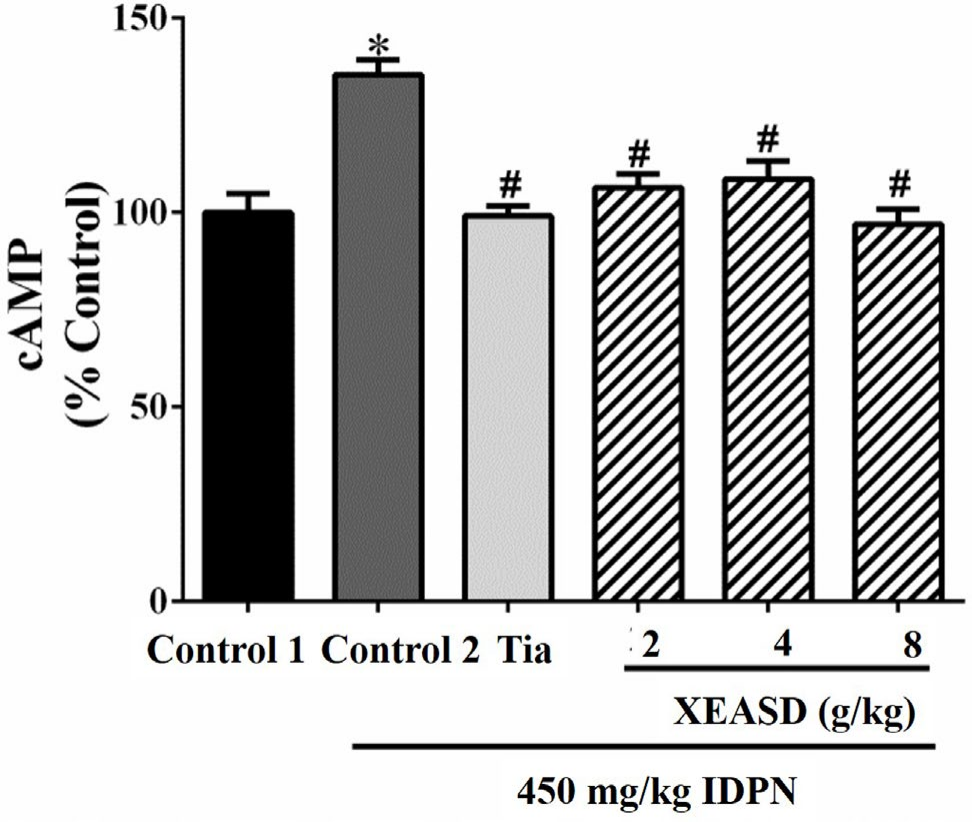
Effects of XEASD treatment on plasma cAMP levels in IDPN-induced TS mice. Biochemical assay of plasma cAMP levels was performed as described in Materials and Methods. Control 1 represents normal control mice; control 2 represents IDPN-challenged control mice. Data are expressed as the percentage of non-challenged control 1 values. *P < 0.05, significantly different from the control 1; ^#^ *P* < 0.05, significantly different from the IDPN control 2. The value of cAMP level of control 1 (mean ± S.E.M.) = 0.50 ± 0.05 (pmol/mg protein).

The effects of Tia and XEASD on the plasma cAMP levels of the mice are shown in **Figure 5**. The result indicated that the IDPN challenge significantly decreased plasma levels of cAMP (29%, *P* < 0.01) in mice. Both Tia and XEASD treatment at a dose of 2 g/kg partially reversed these changes in TS mice to a similar extent (17%, *P* < 0.05) when compared with the IDPN control mice.

#### Phosphorylated-CREB (p-CREB) Levels in Brain Tissue of TS Mice

**Figure 6:**
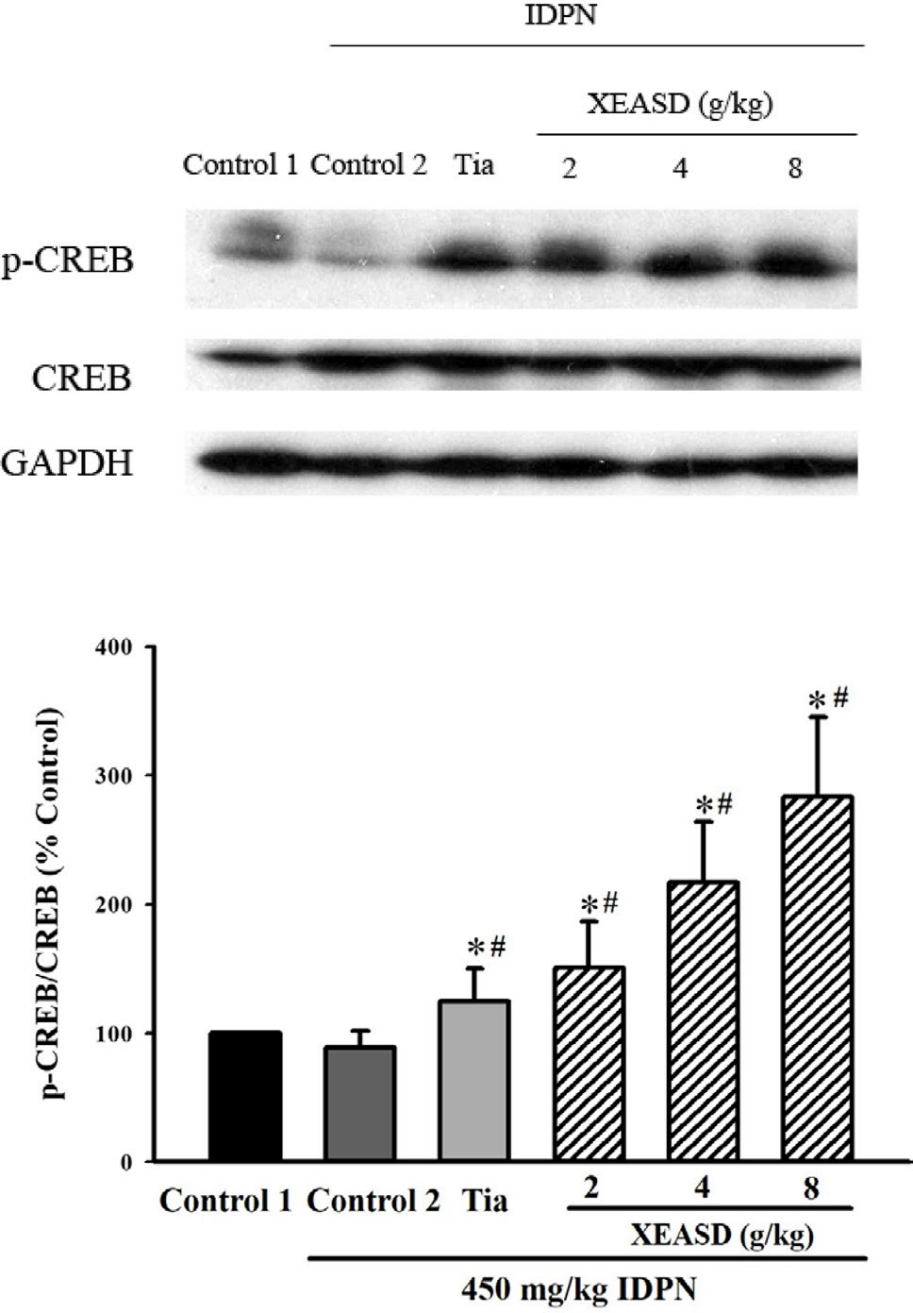
Effects of XEASD treatment on the phosphorylation of CREB in brain tissue of IDPN-induced TS mice. Phosphorylated CREB and total CREB were measured by western blot analysis. Control 1 represents normal control mice; control 2 represents IDPN-challenged control mice. The values (arbitrary units) for phosphorylated CREB were normalized with reference to total CREB levels (arbitrary units) in the samples and expressed in % control 1. *P < 0.05, significantly different from the control 1; ^#^ *P* < 0.05, significantly different from the IDPN control 2.

IDPN challenge did not cause any detectable change in p-CREB levels in mouse brain tissue. Both Tia and XEASD (2–8 g/kg) treatments significantly (and dose-dependently) increased the levels of brain p-CREB (24%, *P* < 0.05 and 51–183%, *P* < 0.01, respectively) in TS mice, when compared with the untreated TS controls (**Figure 6**).

#### Brain Mitochondrial Antioxidant Status

**Figure 7:**
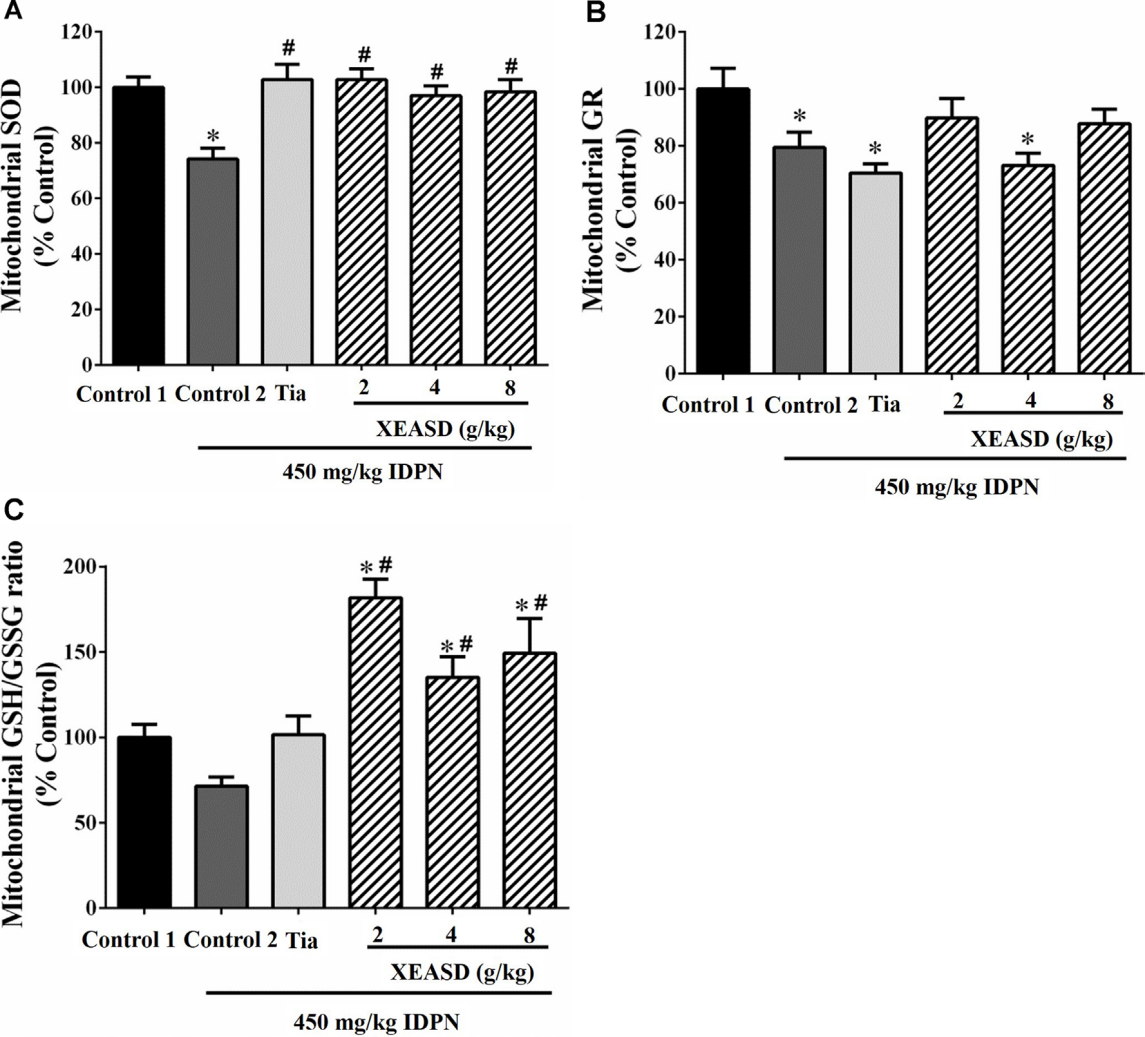
Effects of XEASD treatment on brain mitochondrial antioxidant status in IDPN-induced TS mice. **(A)** Biochemical assay of MnSOD activity was performed as described in Materials and Methods. **(B)** Mitochondrial GR activity was measured as described in Materials and Methods. **(C)** Mitochondrial GSSH/GSSG ratio was measured as described in Materials and Methods. Control 1 represents normal control mice; control 2 represents IDPN-challenged control mice. Data are expressed as the percentage of non-challenged control 1 values. *P < 0.05, significantly different from the control 1; ^#^ *P* < 0.05, significantly different from the IDPN control 2. The value of GSH/GSSG ratio of control 1 (mean ± S.E.M.) was 31.3 ± 2.68. The values of GR activity of control 1 (mean ± S.E.M.) and SOD activity of control 1 (mean ± S.E.M.) were 21.1 ± 1.42 (mU/mg protein) and 5.69 ± 0.66 (U/mg protein), respectively.

While IDPN challenge caused a slight but insignificant decrease in brain mitochondrial GSH/GSSG ratio (an index of antioxidant status) in mice, XEASD (2−8 g/kg) but not Tia treatment produced significant increases in the GSH/GSSG ratio (35−82%, *P* < 0.01) in TS mice, when compared with the untreated TS controls (**Figure 7A**). IDPN challenge significantly decreased brain mitochondrial GR activity (by 20%, *P* < 0.05). XEASD treatment (2 g/kg) partially but not significantly restored GR activity to the control level in TS mice (**Figure 7B**). IDPN challenge significantly suppressed mitochondrial MnSOD activity by 25% (*P* < 0.01). Both Tia and XEASD treatments (2−8 g/kg) completely reversed the inhibitory action of IDPN on mitochondrial MnSOD activity (*P* < 0.01) (**Figure 7C**).

#### Brain Cytosolic GCL Activity

**Figure 8:**
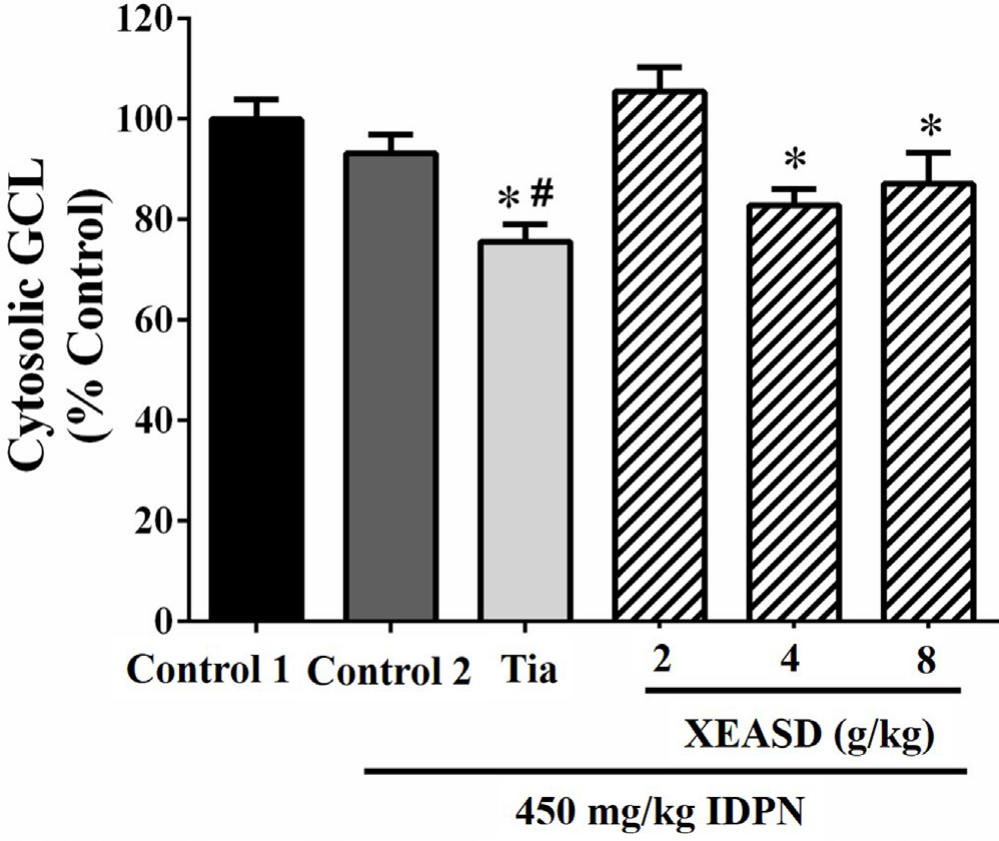
Effects of XEASD treatment on brain cytosolic GCL activity in IDPN-induced TS mice. Biochemical assay of cytosolic GCL activity was performed as described in Materials and Methods. Control 1 represents normal control mice; control 2 represents IDPN-challenged control mice. Data are expressed as the percentage of non-challenged control 1 values. *P < 0.05, significantly different from the control 1; ^#^ *P* < 0.05, significantly different from the IDPN control 2. The value of GCL activity of control 1 (mean ± S.E.M). was 73.4 ± 5.29 (mU/mg protein).

**Figure 8** shows that, whereas IDPN did not produce any detectable change in the brain cytosolic GCL activity in mice, Tia treatment caused small but significant decreases in cytosolic GCL activity in TS mice (19%, *P* < 0.05). XEASD treatment at a dose of 2 g/kg slightly but insignificantly increased the GCL activity in IDPN-challenged mice.

## Discussion

In the present study, IDPN, which is a neurotoxin (Morandi et al., 1987), was used to produce the mouse model of TS. IDPN challenge significantly increased the stereotypy score and locomotor activity in mice, with the production a persistent behavioral syndrome mimicking TS (Zhang and Li, 2015). Tia, a selective D2R (dopamine receptor) antagonist, has been shown to antagonize the dyskinetic oro-linguo-facial symptoms triggered by the injection of DA in the striatum. Both clinical and experimental studies demonstrated that Tia possesses anti-tic effects in both TS children and apomorphine-induced TS in experimental animals (Bock et al., 2004; Eddy et al., 2011). Thus, Tia was used as a positive control drug for comparison with XEASD in protecting against TS. Our results showed that treatment with Tia or XEASD ameliorated the severity of behavioral symptoms in TS mice, as evidenced by significant decreases in stereotypy score and locomotor activity.

Abnormal levels of amino acid-related neurotransmitters, such as GABA and glutamate, which are involved in the CSTS circuits, have been shown to be associated with the development of TS. GABA is the primary inhibitory neurotransmitter of medium spiny neurons in the striatum and interneurons located in both the striatum and cortex. GABAergic neurons produce GABA, which is released at synapses formed between GABAergic neurons and target cells. In Tourette patients, the involvement of striatal GABAergic neurons was indicated by a reduced number of parvalbumin-positive GABAergic interneurons in the striatum (Kataoka et al., 2010) and a decreased binding of GABA-A receptors in *postmortem* brains of TS patients (Lerner et al., 2012), which was also associated with a reduced level of brain GABA. However, our results showed that the IDPN challenge caused a significant increase in striatal GABA levels in mice when compared with the control group. Although our finding is contradictory with that of the aforementioned observation in *postmortem* human brains, it is consistent with the finding of Zhang *et al*., in which the levels of GABA in the striatum were increased in IDPN-treated mice (Zhang et al., 2014). The increase in GABA levels in the striatum, as observed in the present and other studies, is possibly due to an adaptive response to suppress the hyperactivity that causes the repetitive and involuntary movements and noises in TS mice. In an animal study investigating the relationship between GABA levels and brain structure, it was observed that elevated GABA levels are associated with an increased number of connecting fibers in the corpus callosum, a structure connecting the two hemispheres of the brain. It was hypothesized that the increase in connecting fibers positively correlated with an increased number of neuronal signals produced, which necessitated a higher level of GABA to counteract the excess hyperactivity (Draper et al., 2014). In this connection, the alleviation of symptoms of tics and involuntary movements in TS mice treated with Tia or XEASD was paralleled by significant decreases in GABA levels in the striatum.

In our previous study, we did not examine the effect of XEASD on the levels of glutamate and aspartate in PC12 cells. While in the current study, we demonstrated that IDPN TS mice showed a slight decrease in glutamate levels in brain tissues; both Tia and XEASD treatments greatly increased brain glutamate levels in these animals. Mahone et al. (2018) have demonstrated that in children with TS, increased premotor cortex glutamate levels were closely paralleled by an improved selective motor inhibition. XEASD treatment also increased levels of aspartate in brain tissues of TS mice. Given that glutamate and aspartate play important metabolic roles in neurons, their elevated levels in the brain may implicate their role in metabolic function in addition to neurotransmission. Furthermore, the increases in glutamate and aspartate levels may reflect other compensatory neuropsychological functions such as a reduction of motor overflow. Therefore, the elevation of glutamate and aspartate levels by XEASD in the brain may contribute to the inhibition of abnormal motor function in TS mice. Abnormalities in second messenger systems have been proposed as an underlying pathogenetic mechanism of TS (Singer et al., 1995). As observed in the present study, XEASD treatment was found to reverse the decreased plasma cAMP levels in TS mice, which suggests an improvement in the cAMP second messenger system.

A recent study has demonstrated that pre-incubation of PC12 cells with XEASD can stimulate neuronal differentiation, as evidenced by an increase in the expression of NF 160 and NF 200 proteins (Li et al., 2018). The finding that XEASD induced the phosphorylation of CREB suggested the involvement of the PKA-CREB signaling pathway in its pharmacological action. The possibility has been raised that altering brain functioning and architecture during a sensitive phase of neural development may play an important role in the development of TS (Nespoli et al., 2016). The results obtained in the present study also showed that both Tia and XEASD treatments increased the phosphorylation of CREB in brain tissues of TS mice. This observation may indicate the possible induction of neural differentiation by XEASD in children with TS, with a resultant alleviation of TS symptoms. The stimulation of the CREB pathway by XESAD suggests the possibility that XEASD may be effective in treating obsessive compulsive disorder, which occurs in a large percentage of patients with TS (Buzan et al., 2000; Como et al., 2005; Arora et al., 2013).

Oxidative stress has been associated with the pathogenesis of TS. In this regard, XEASD has been shown to activate the antioxidant response element in PC12 cells, resulting in an enhancement in the expression of antioxidant defense genes and thereby trigger a cellular antioxidant response (Li et al., 2018). In the present study, the effect of XEASD treatment on cellular redox status in brain tissues was examined in TS mice. To cope with oxidative stress, cells are equipped with sophisticated endogenous antioxidant systems, which include non-enzymatic antioxidants, such as GSH and alpha-tocopherol (or vitamin E), and enzymatic antioxidants, such as MnSOD, catalase, glutathione peroxidase, and heme oxygenase (Birben et al., 2012; Bajpai et al., 2017). Among these, the glutathione redox system is regarded as the first line of defense against ROS (Chen and Ko, 2013) and, as such, the GSH/GSSG ratio has been used to assess the redox status in biological systems. A reduction in the GSH/GSSG ratio appears to correlate with the proliferating state of the cell, and an increasingly more oxidized state is characteristic of cells during differentiation and apoptosis. Our findings showed that treatment with XEASD significantly increased the mitochondrial GSH/GSSG ratio in mouse brains, indicative of a glutathione-dependent antioxidant response. In order to elucidate the mechanism underlying this action, the activities of GR and GCL, which are responsible for the regeneration and biosynthesis of GSH, respectively (Quintana-Cabrera et al., 2012), were also measured. The results indicated that the enhancement of glutathione antioxidant status may be mainly due to the increased regeneration of GSH from GSSG, rather than an increase in the biosynthesis of GSH in XEASD-treated mice. In addition, the increase in MnSOD activity by XEASD treatment may further increase antioxidant capacity in mouse brain tissue. Since mitochondria are the major source of ROS in neurons, the elevation in glutathione-dependent antioxidant response as well as MnSOD activity may also enhance mitochondrial functions in XEASD-treated mouse brains, such as ATP generation. ATP is necessary for vesicle transport and neurotransmitter release as well as the activation of downstream signaling pathway following the binding of neurotransmitters to receptors (Vos et al. 2010). Therefore, the ability of XEASD treatment to alter the neurotransmitter levels may be related to the changes in antioxidant status in brain tissues of TS mice.

## Conclusion

In conclusion, XEASD treatment has been shown to be effective in ameliorating the severity of behavioral symptoms of TS in an experimental mouse model of the clinical disorder. This beneficial effect was associated with increases in glutamate and aspartate levels in brain tissue, as well as a decrease in striatal GABA levels. XEASD treatment also increased brain p-CREB levels in TS mice. In addition, XEASD treatment enhanced brain mitochondrial antioxidant status in affected animals. Taken together, results obtained from the present study provide an insight into the pharmacological basis for the beneficial effects of XEASD treatment of TS in children.

## Ethics Statement

All experimental protocols were reviewed and approved by the University Ethics Committee on Animal Research Practice at the Hong Kong University of Science and Technology.

## Author Contributions

All authors listed have made substantial, direct, and intellectual contribution to the work and approved it for publication.

## Funding

This work was supported by the National Natural Science Foundation of China (Grant No. 81603356), National Natural Science Foundation of China (81804052), Natural Science Foundation of Guangdong Province (2018A030313305), and Shenzhen Science and Technology Plan Project (ZDSYS201606081515458).

## Conflict of Interest Statement

The authors declare that the research was conducted in the absence of any commercial or financial relationships that could be construed as a potential conflict of interest.

The handling Editor declared a shared affiliation, though no collaboration, with three of the authors, ZL, JQ, and JC, at the time of review.
